# Supplementary figures and images for: Human LAMP1 accelerates Lassa virus fusion and potently promotes fusion pore dilation upon forcing viral fusion with non-endosomal membrane
Source: PLoS Pathog. 2022 Aug 15;18(8):e1010625. doi: 10.1371/journal.ppat.1010625 (PMC9410554; doi:10.1371/journal.ppat.1010625)

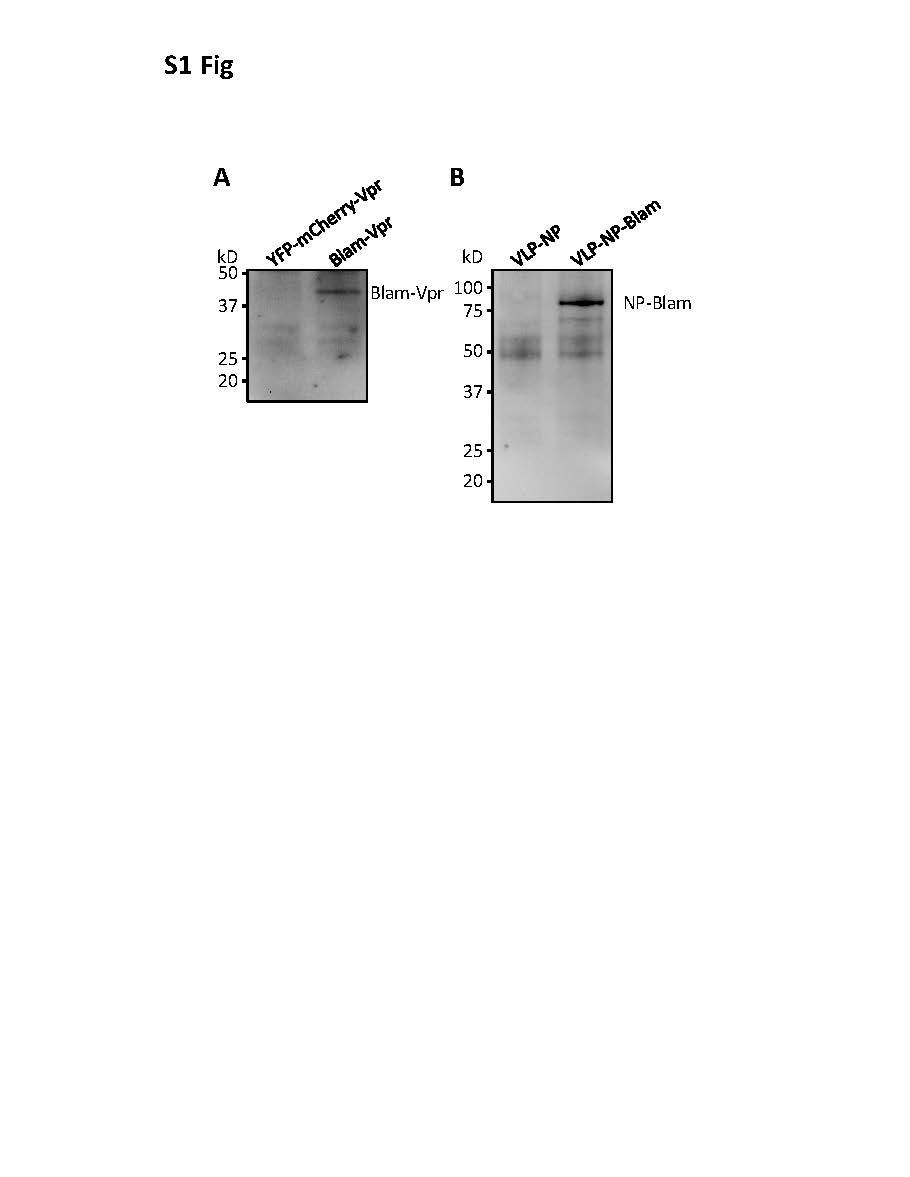

Supplement: S1 Fig — BlaM-Vpr (A) or NP-BlaM (B) in LASVpp-BlaM and LASV-VLP-BlaM particles, respectively, were examined by Western blotting using anti-β-lactamase antibody. LASVpp carrying mCherry-YFP-Vpr and LASV-VLP-NP were used as negative controls for non-specific with signal. (JPG) [file ppat.1010625.s001.jpg]

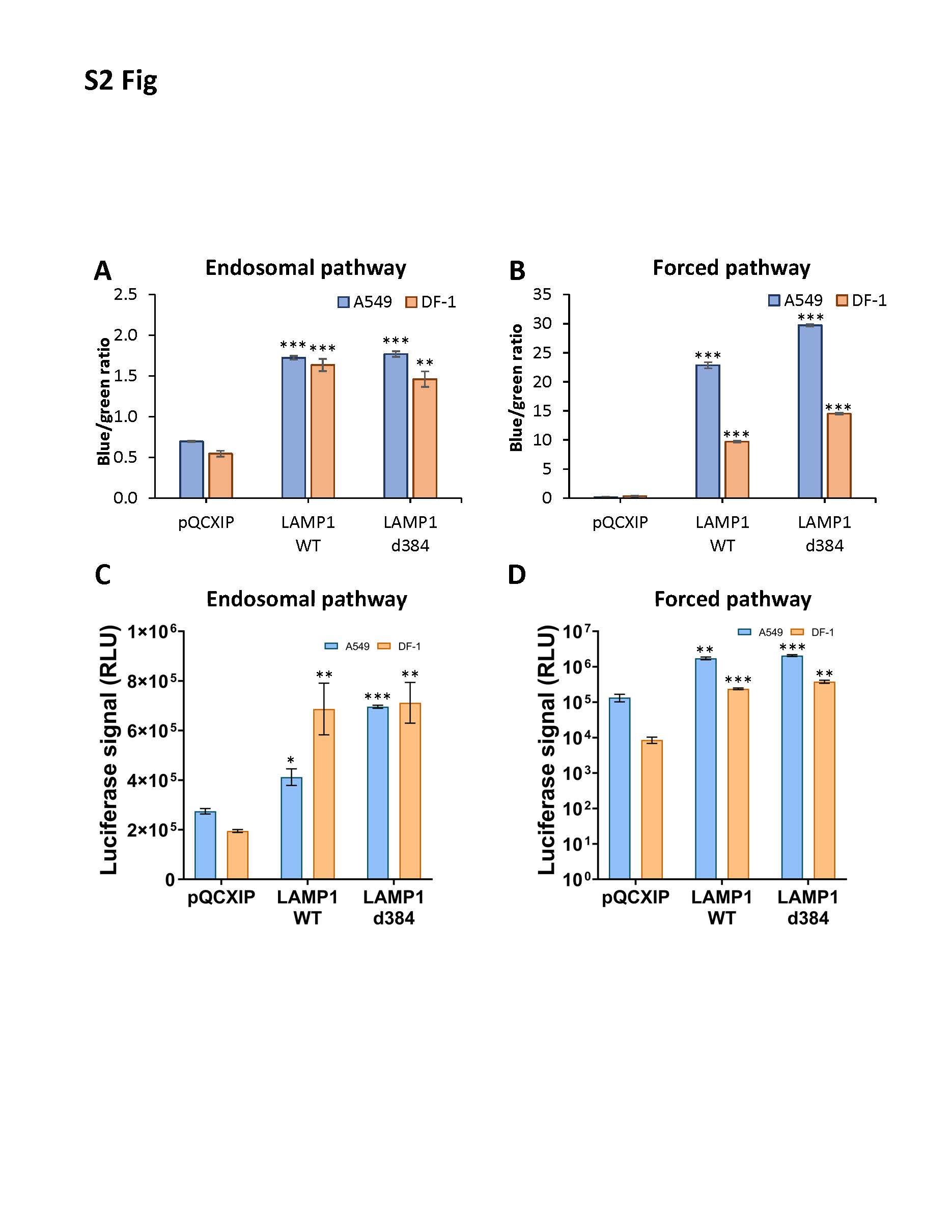

Supplement: S2 Fig — Raw data from representative experiments are shown. (A) LASVpp-BlaM fusion with A549 and DF-1 cells. Due to the limited dynamic range of the BlaM assay, A549 and DF-1 cells were infected with MOI of 0.1 and 0.05, respectively, to ensure a linear range of the BlaM signal (blue/green fluorescence ratio). (B) Low pH-forced fusion of LASVpp with A549 and DF-1 cells. MOI of 5, 0.5 and 0.05 were used for A549-pQCXIP, DF-1-pQCXIP and for A549 or DF-1 cells expressing LAMP1-WT or LAMP1-d384, respectively. The plotted signal in panel (A) and (B) is corrected for the dilution factor. (C) LASVpp infection of A549 and DF-1 cells. MOI of 1 and 10 were used for A549 and DF-1 cell respectively. (D) LASVpp infection through low pH bypass protocol in A549 and DF-1 cells. MOI of 0.1 and 10 were used for A549 and DF-1 cell respectively. Data in panel (D) are plotted in logarithmic scale. Data shown are means ± SD of three technical replicates. Statistical significance was determined by Student’s t-test. *, p<0.05; **, p<0.01; ***, p<0.001. Asterisks on the top of bars represent significance relative to the vector control. (JPG) [file ppat.1010625.s002.jpg]

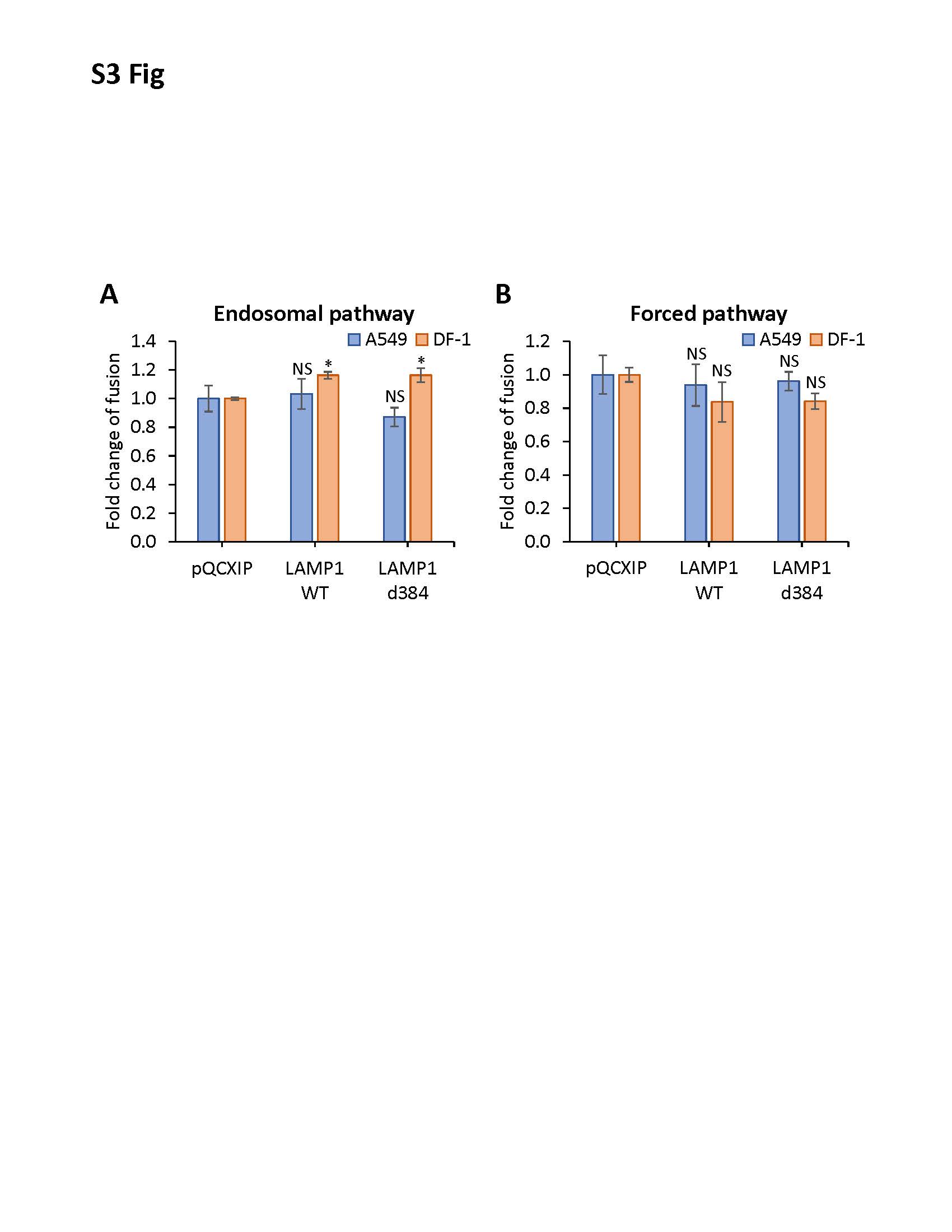

Supplement: S3 Fig — (A) VSVpp-BlaM pseudovirus fusion with A549 and DF-1 cells. VSVpp entry through an endosomal pathway was initiated by pre-binding pseudoviruses in the cold, shifting to 37°C and incubating for 2 h. (B) Low pH-forced fusion of VSVpp with A549 and DF-1 cells. Cells were pretreated with 0.2 μM BafA1 for 1 h prior to binding pseudoviruses in the cold. Fusion was triggered by applying pH 5.0 citrate buffer at 37°C for 20 min followed by additional incubation at neutral pH, 37°C for 30 min. Data are means ± SD of three technical replicates of a representative experiment. Statistical significance was determined by Student’s t-test. *, p<0.05; NS, not significant. Asterisks on the top of bars represent significance relative to the vector control. (JPG) [file ppat.1010625.s003.jpg]

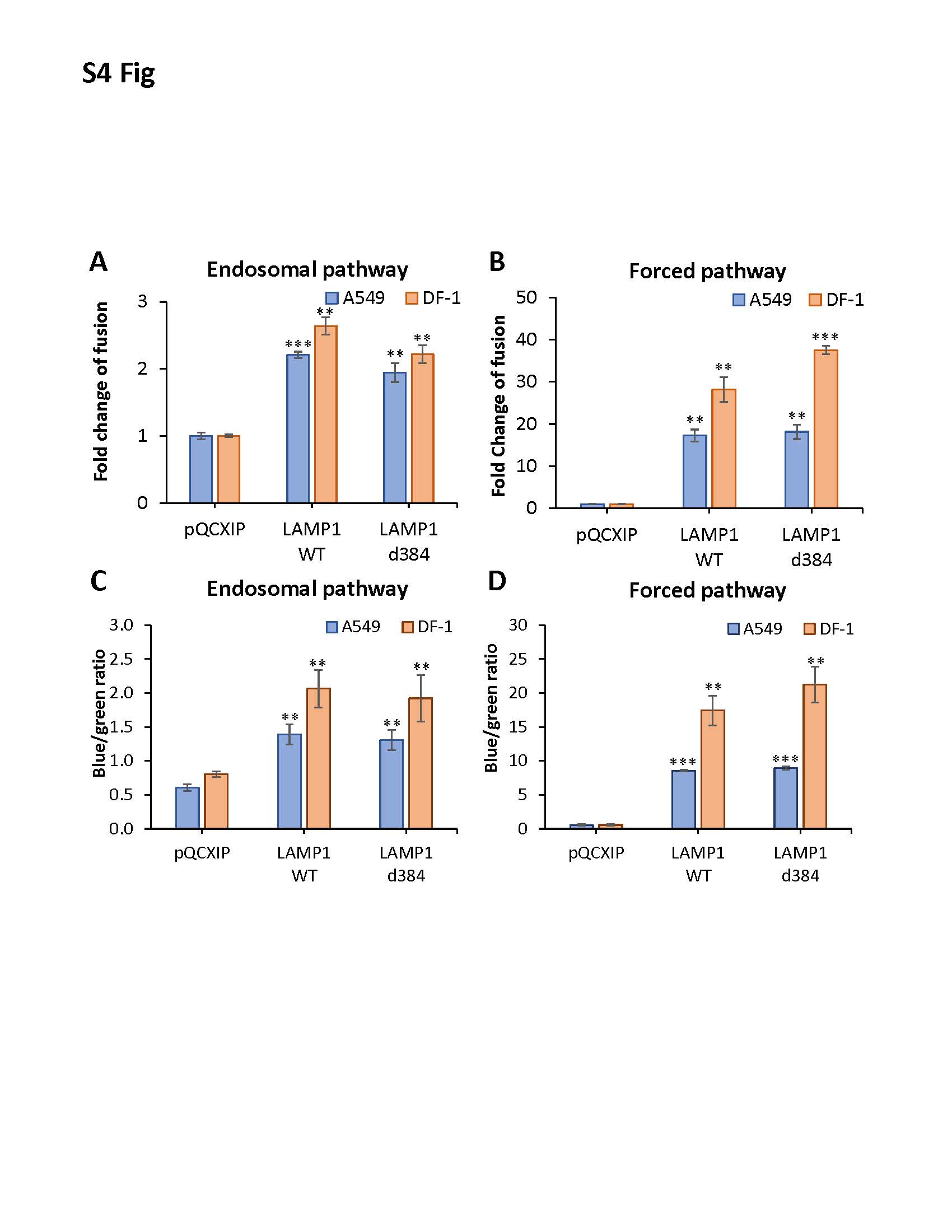

Supplement: S4 Fig — (A) LASV-VLP-BlaM fusion with A549 and DF-1 cells. LASV-VLP entry through an endosomal pathway was initiated by pre-binding the VLP in the cold, shifting to 37°C and incubating for 2 h. (B) Low pH-forced fusion of LASV-VLP with A549 and DF-1 cells. Cells were pretreated with 0.2 μM BafA1 for 1 h prior to binding the VLPs in the cold. Fusion was triggered by applying pH 5.0 citrate buffer at 37°C for 20 min followed by additional incubation in a neutral pH medium at 37°C for 30 min. (C) Representative raw data of LASV-VLP fusion with A549 and DF-1 shown in panels (A). (D) Representative raw data of LASV-VLP low pH-forced fusion results shown in panels (B). Due to the limited dynamic range of the BlaM assay, different dilutions of the virus stock were used to ensure a linear range. For LASV-VLP-BlaM entry through endosomal pathway, 2x and 3x less VLPs were used to infect hLAMP1-expressing cells than control A549 and DF-1 cells, respectively. For the forced fusion of LASV-VLP-BlaM, 3x and 50x less VLPs were used to infect LAMP1 expressing cells than control A549 and DF-1 cells, respectively. Data shown in panels (A) and (B) are means ± SD of three independent experiments. Data shown in panel (C) and (D) are means ± SD of three technical replicates. Data were analyzed by Student’s t-test. **, p<0.01; ***, p<0.001. (JPG) [file ppat.1010625.s004.jpg]

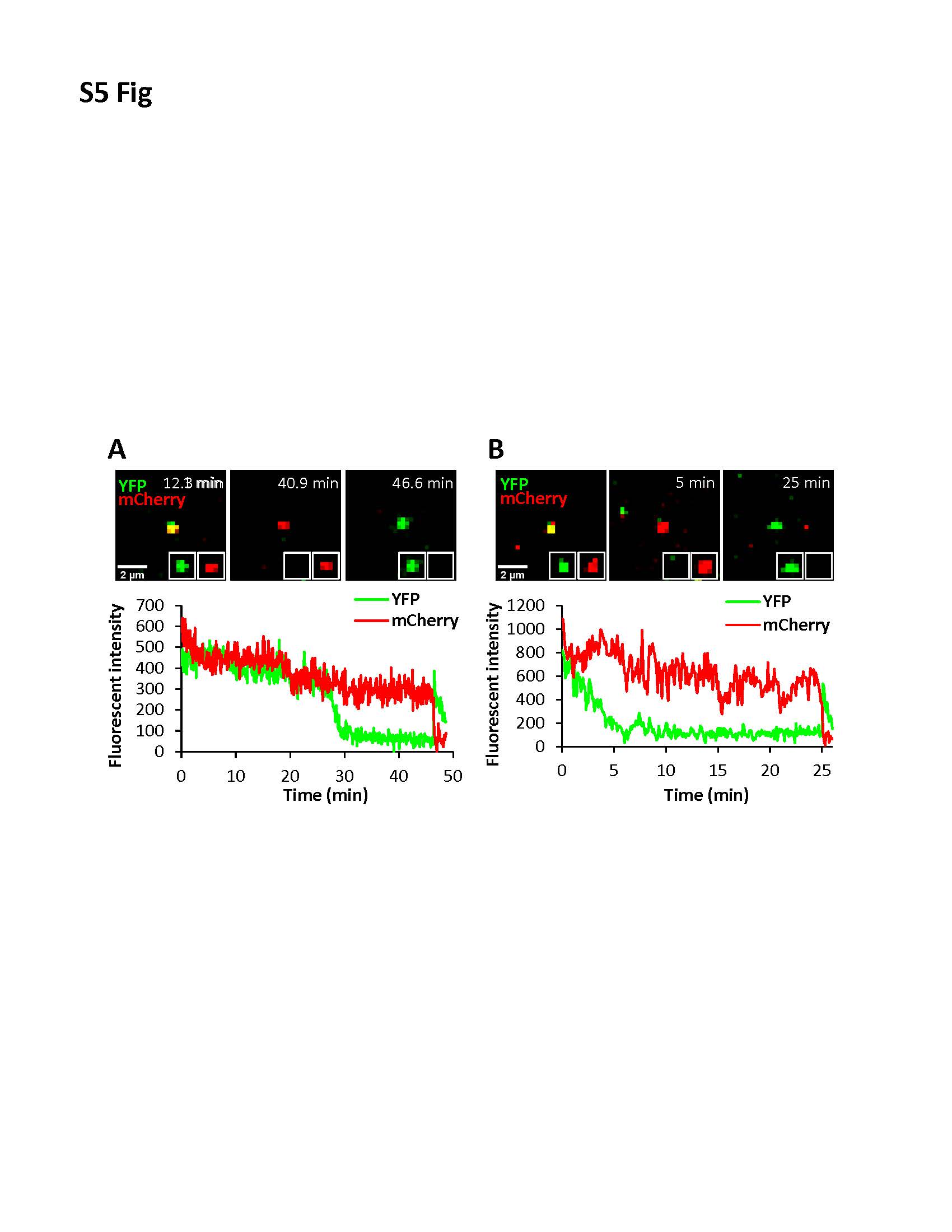

Supplement: S5 Fig — (A) Time lapse images (top) and fluorescence traces (bottom) of single LASVpp fusion with a DF-1-pQCXIP cell showing YFP quenching at 29.5 min and YFP dequenching/mCherry loss at 46.5 min corresponding to virus interior acidification and fusion, respectively. (B) Time lapse images (top) and fluorescence traces (bottom) of single LASVpp fusion with a DF-1-LAMP1-d384 cell showing YFP quenching at 5 min and YFP dequenching/mCherry loss at 25 min, indicating virus interior acidification and fusion, respectively. (JPG) [file ppat.1010625.s005.jpg]

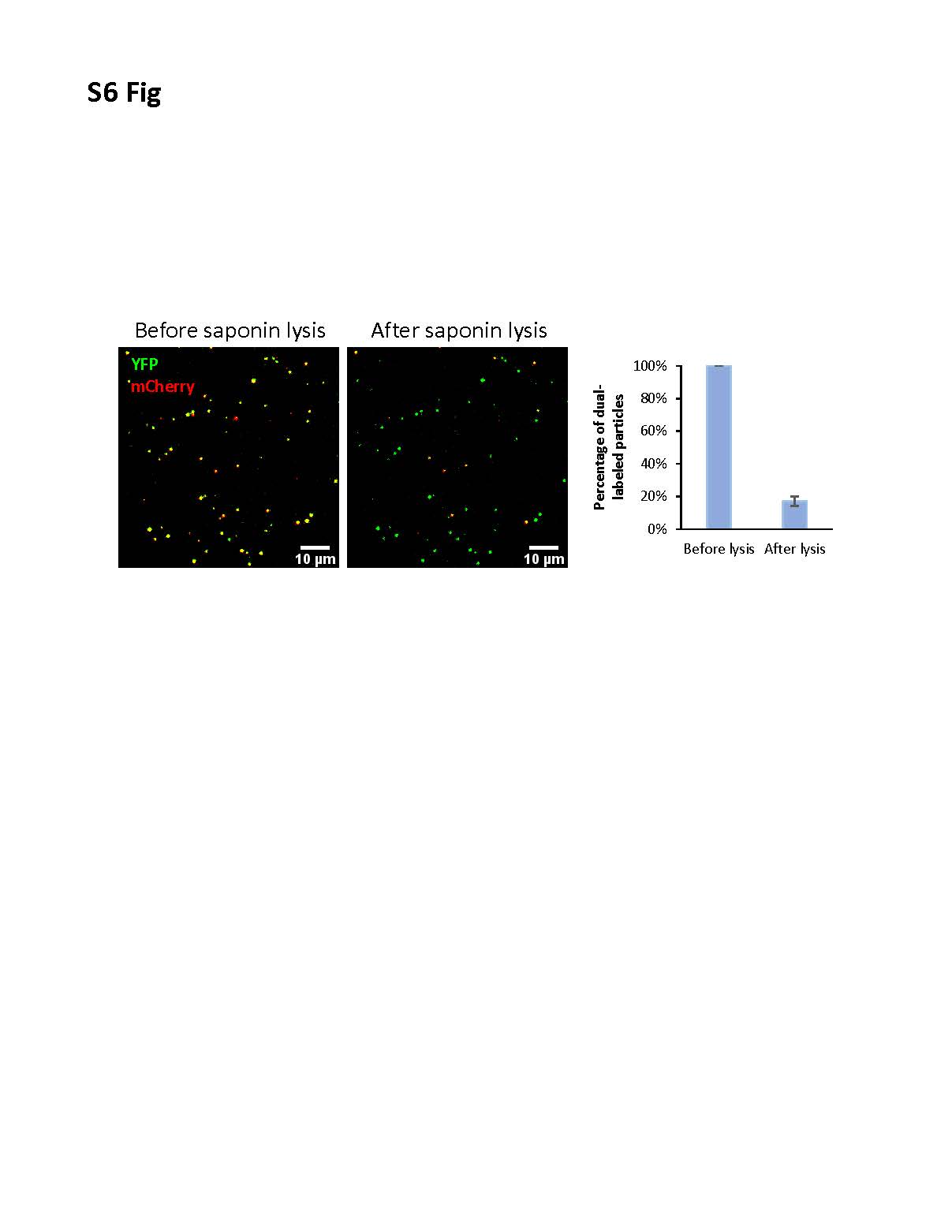

Supplement: S6 Fig — Images (left panel) and quantification (right panel) of virus lysis (loss of mCherry from YFP-Vpr labeled particles) before and 10 min after application of saponin. A small fraction (~15%) of immature HIV-1 pseudoviruses retained mCherry. Data are means ± SD from 4 image fields analyzed. (JPG) [file ppat.1010625.s006.jpg]

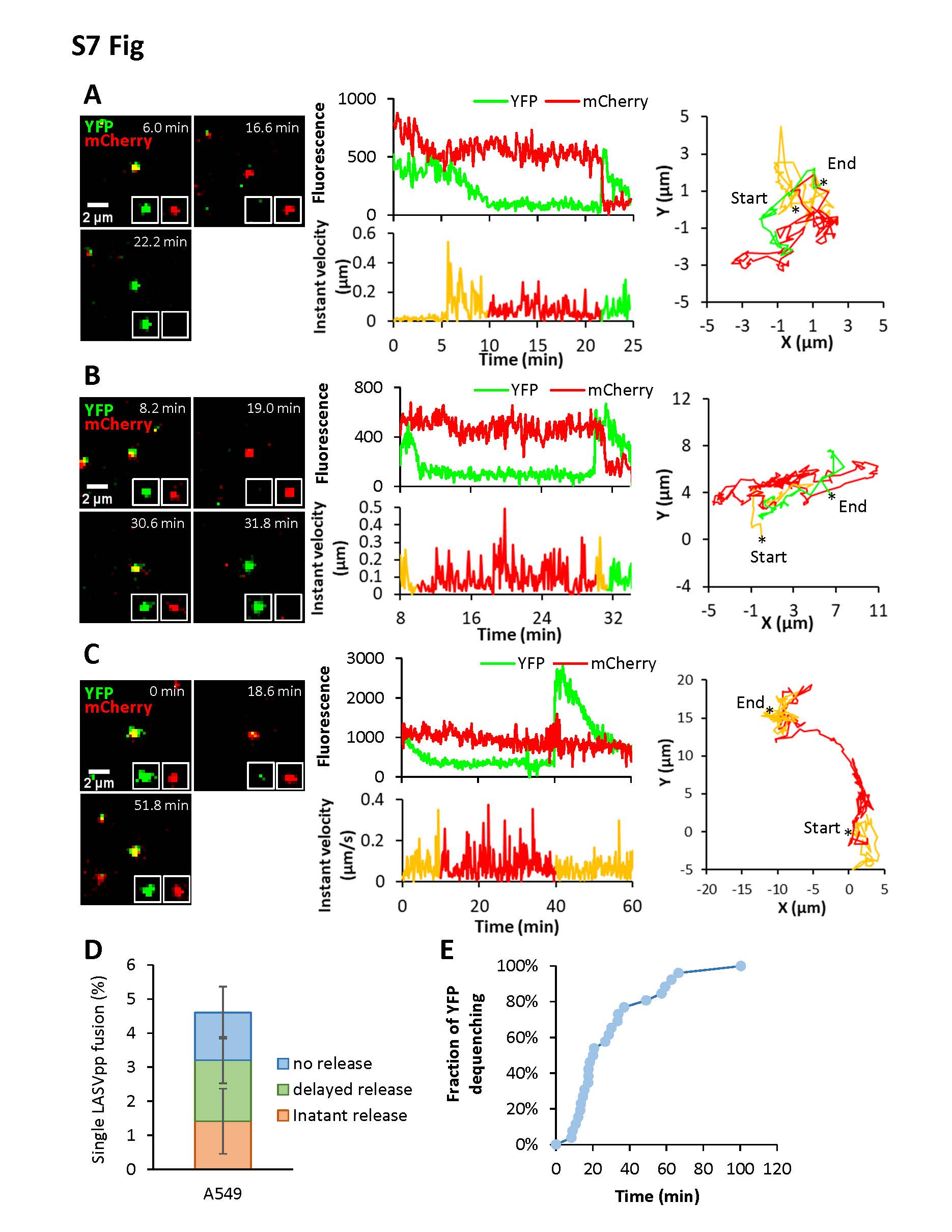

Supplement: S7 Fig — (A) A representative LASVpp fusion event (YFP dequenching) with concomitant mCherry release (quick fusion pore dilation). (B) LASVpp fusion event with delayed mCherry release relative to YFP dequenching. (C) LASVpp fusion event (YFP dequenching) without mCherry release. For all panels, time lapse images (left), fluorescence intensity traces (middle top), instant velocity (middle bottom) and trajectory (right) are shown. (D) Fraction of LASVpp fusion events according to the pore enlargement phenotype (instant and delayed mCherry release or lack of mCherry release). Data are means ± SD of 3 independent experiments. (E) Kinetics of nascent pore formation (YFP dequenching) for single LASVpp fusion. (JPG) [file ppat.1010625.s007.jpg]

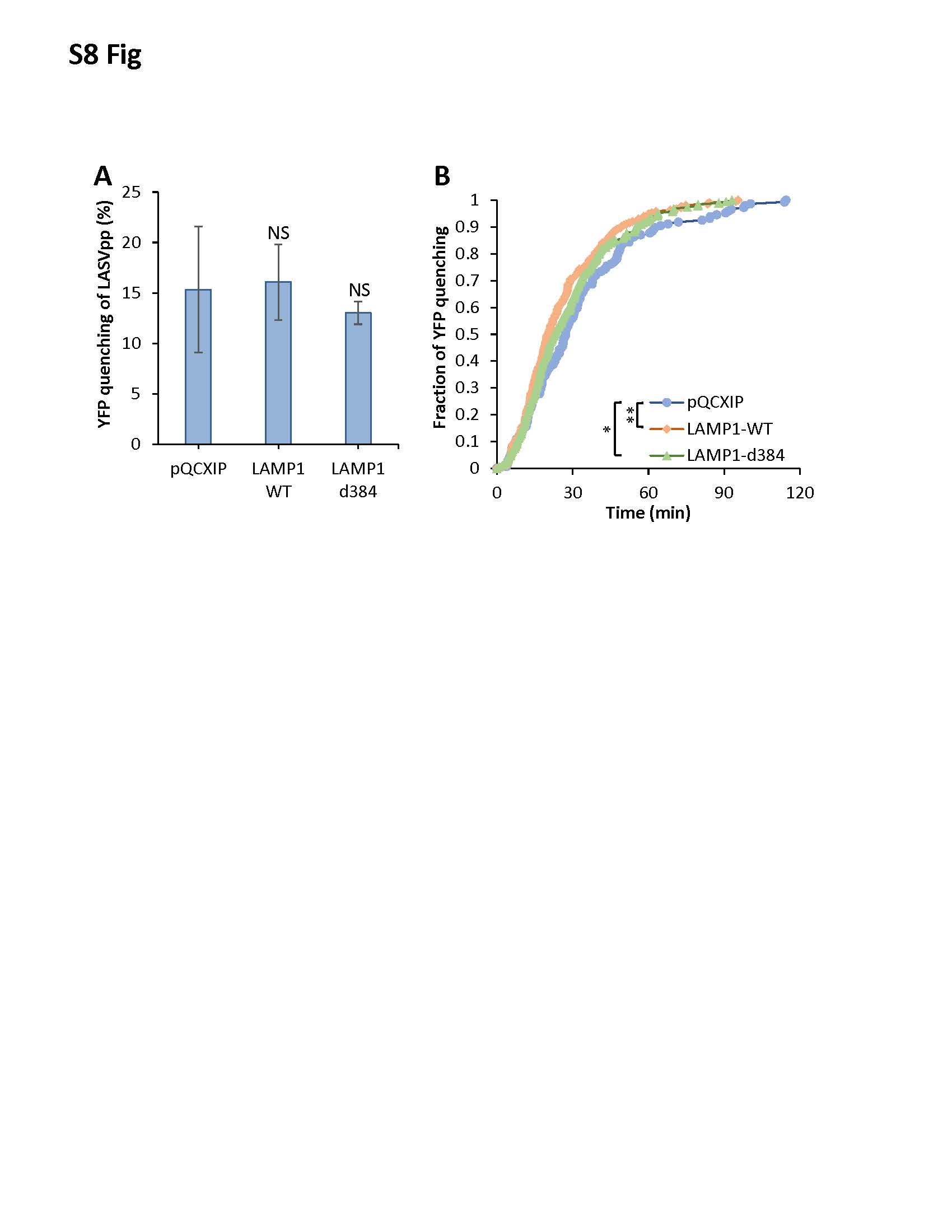

Supplement: S8 Fig — (A) Fraction of single LASVpp exhibiting YFP quenching in DF-1 pQCXIP, LAMP1-WT and LAMP1-d384 cells. Data shown are means ± SD of 5 independent experiments. (B) Kinetics of the YFP quenching of single LASVpp in control and hLAMP1 expressing DF-1 cells. Data were analyzed by Student’s t-test. *, p<0.05; **, p<0.01; NS, not significant. (JPG) [file ppat.1010625.s008.jpg]

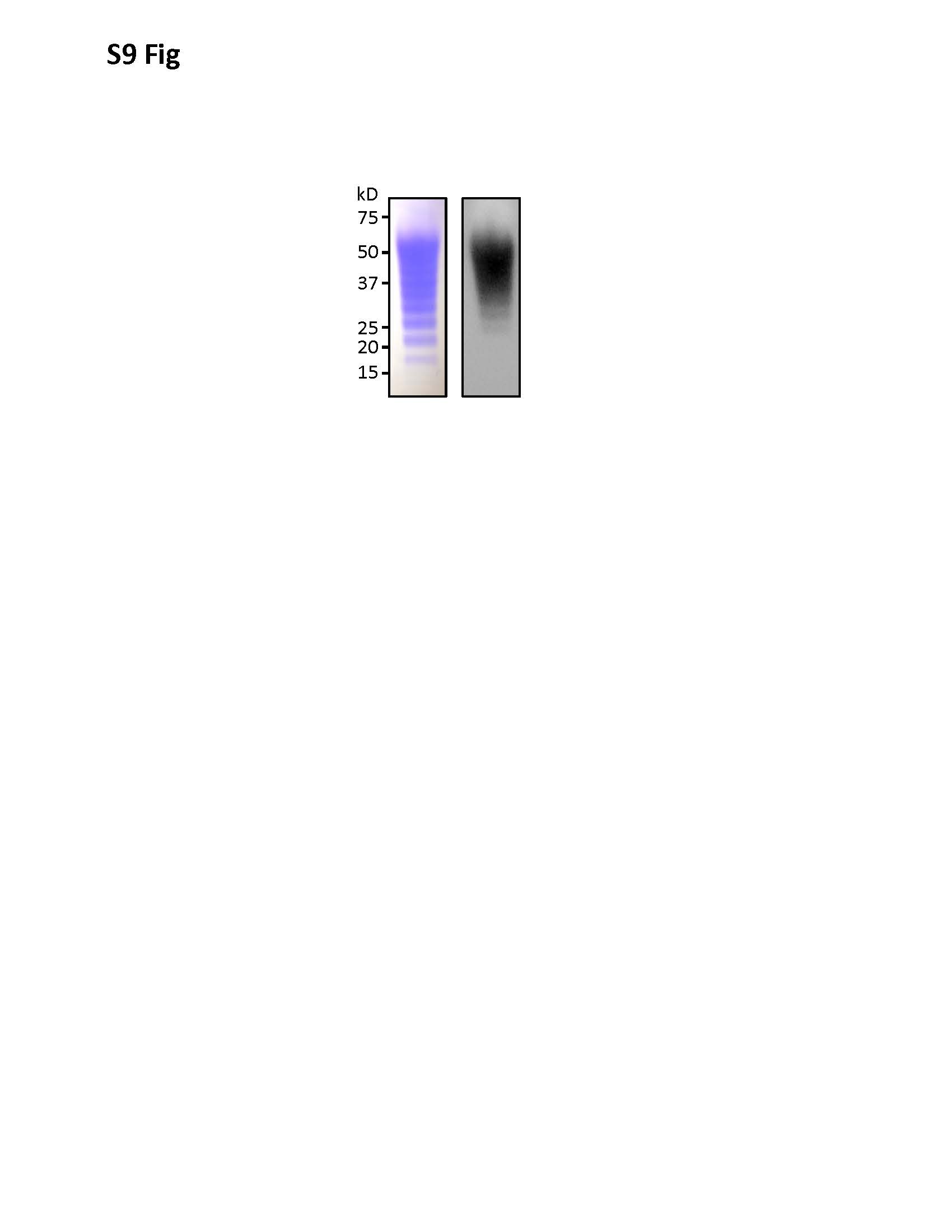

Supplement: S9 Fig — Purified sLAMP1 were detected using Coomassie Blue staining (left) and verified by SDS-PAGE and Western-blotting using anti-hLAMP1 antibody (right). (JPG) [file ppat.1010625.s009.jpg]

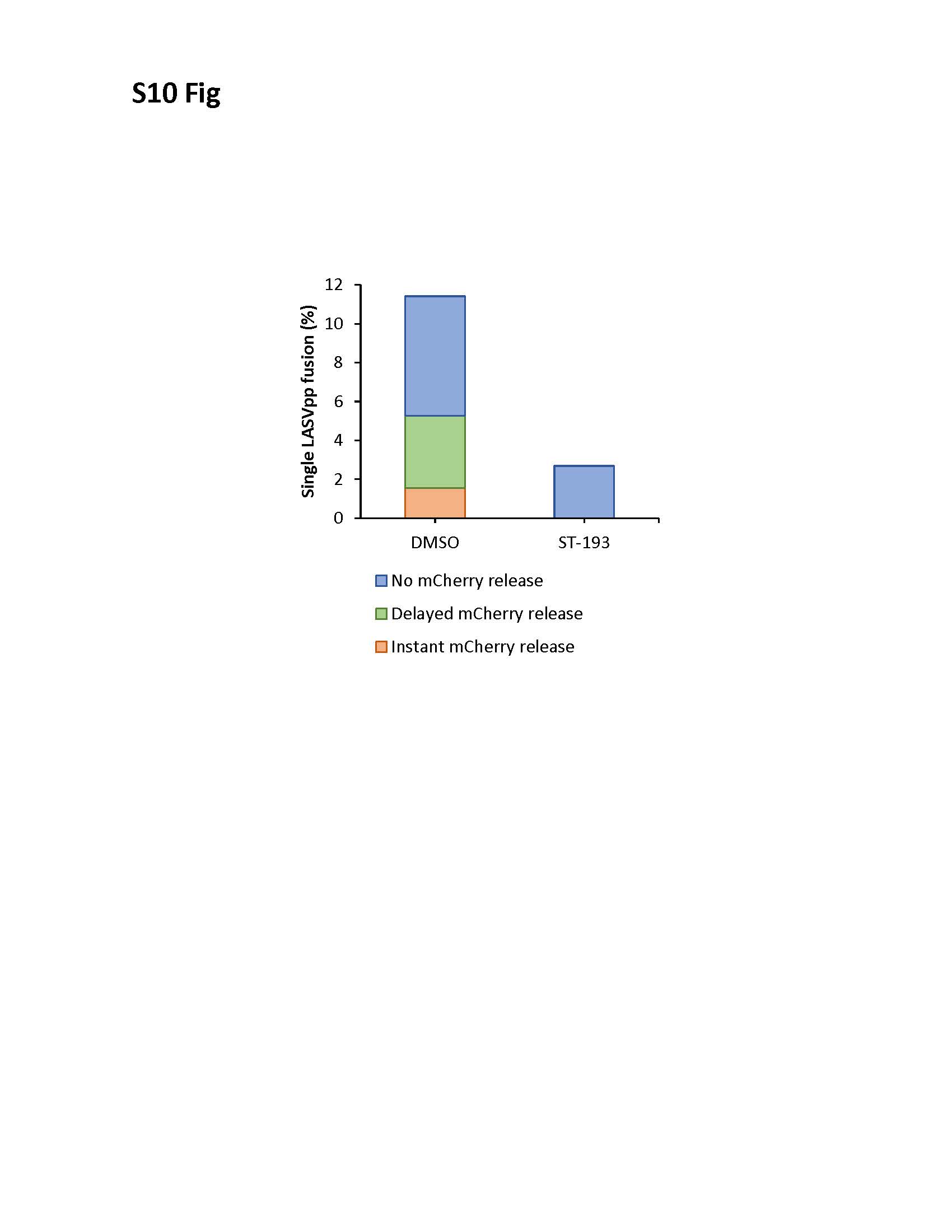

Supplement: S10 Fig — LASVpp were bound to DF-1 cells in the cold, in the presence of 200 μg/ml soluble LAMP1 with or without 10 μM of ST-193. Single LASVpp fusion with the plasma membrane was initiated by addition of 2 ml of warm pH 5.0 citrate buffer supplemented with 200 μg/ml sLAMP1 with or without 10 μM of ST-193. Graph show efficiencies of low pH-forced single LASVpp fusion events with instant mCherry release, delayed mCherry release and without mCherry release with DF-1 cells in the absence of sLAMP1 with or without ST-193. Data shown result of 1 independent experiment. (JPG) [file ppat.1010625.s010.jpg]
