# Supplementary material for: Human LAMP1 accelerates Lassa virus fusion and potently promotes fusion pore dilation upon forcing viral fusion with non-endosomal membrane
Source: PLoS Pathog. 2022 Aug 15;18(8):e1010625. doi: 10.1371/journal.ppat.1010625 (PMC9410554; doi:10.1371/journal.ppat.1010625)
Supplement: Attachment — Submitted filename: LASV-LAMP1_PLoSPath_rebuttal(2).docx [file ppat.1010625.s017.docx]

**Reviewer #1: No critiques**

Response: We thank Reviewer #1 for a very positive evaluation of our manuscript.

**Reviewer #2:**

**Minor issues:**

1) Can finding that hLAMP1 expression does not significantly alter the relative weights of the three types of fusion events: instant, delayed pore opening, no dilation (Fig. 5A) indicate that Lamp1 influences the stage(s) preceding the fusion pathway? For instance, can you exclude the effects of the LAMP1 expression at the surface of the cells beyond the relatively low endogenous levels of this receptor in A549 cells, Fig. 1C on cell surface binding of GPC carrying pseudoviruses?

Response: That is a good point. However, as shown in the figure included below, hLAMP1 expression does not affect LASVpp binding. In addition, the current Fig. S8 shows no notable differences in LASVpp uptake by cells expressing or lacking hLAMP1. Nevertheless, we cannot rule out the possibility that ectopic LAMP1 expression alters endosomal trafficking and endosome acidity.

2) I am somewhat unclear on the relative effects of overexpression of WT LAMP1 that is almost absent at the plasma membrane and LAMP1 d384 that is mostly expressed at the plasma membranes. Can you please further discuss why in Fig. 2B WT LAMP1 and LAMP1 d384 similarly (even if the difference is statistically significant) promote fusion in endosomes and at the plasma membrane?

Response: We are also perplexed by the lack of large differences and can only speculate as to the causes for this. We think that endogenous low levels of hLAMP1 may be sufficient for optimal LASV fusion with endosomes. For the forced fusion, the difference between LAMP1 WT and LAMP1 d384 are more apparent.

3) I am intrigued by the finding that pseudoviruses exposed to acidic pH on the cell surface can still be internalized and fuse with neutral endosomes. Does this outcome (fusing in endosome rather than at the plasma membrane) correlate with how fast these virions are internalized? If fusion cannot happen faster than within certain time and if the virus happens to be internalized within this time, fusion will happen only in endosome.

Response: That’s a good point. Indeed, once conformational changes in the viral glycoprotein are triggered at the cell surface, the sites of fusion (plasma membrane vs endosome) is likely determined by the competition between the virus fusion and uptake kinetics. We would like to point out, however, that we are not explicitly measuring virus endocytosis here and cannot be certain if forced LASVpp fusion does occur in endosomes.

4) To distinguish direct effects of LAMP1 (as a receptor) from indirect effects such as domain organization in PM one may ask whether LAMP1 expression influences fusion for viral fusogens that do not utilize LAMP1 as a receptor, say for HIV Env?

Response: We thank the reviewer for this excellent suggestion. The revised manuscript includes the new Fig. S3 showing that hLAMP1 expression does not significantly affect the endosomal or forced fusion of VSV-G pseudotyped particles.

5) I suggest the sudden appearance of the term ‘type II fusion’ needs to be preceded by a brief explanation of the way you have classified single virus fusion events into Type 1 and 2 in Ref 20. Also is there any evidence to suggest that fusion is dependent on pre-fusion permeabilization? The authors show that LASVpp undergoes type II fusion independent of a target cell (A549 and DF-1 cells) and irrespective of whether fusion occurred in endosomes or at the plasma membrane. Can pre-fusion permeabilization be uncoupled from fusion in low pH-forced fusion model by using less acidic pH than pH 5.0.

Response: These are all great questions. We have further discussed type II fusion events in our revised manuscript. As to the dependence of fusion on viral membrane permeabilization and the ability to uncouple these events, this is an area of active investigation in the lab for the last two years. We hope to publish in the future these studies, which we consider to be beyond the scope of the present work..

6) Fig. 2D is referred to before Fig. 2C.

Response: This is a fair point, but we prefer not to change the panel arrangement in Fig. 2.

7) The sentence “We have previously shown that hLAMP1 overexpression allows LASV GPC-mediated fusion to occur at higher pH [10, 12].” should be edited, since Ref. 10 is published by another group.

Response: Corrected. Thank you.

**Reviewer #3:**

**Major issues:**

1) It is not surprising that the detailed quantitative effects of different manipulations would have different outcomes for different virus systems or in different cell lines, and there are many possible reasons for that, but I find the early discussion of each of the results too speculative and distracting because it comes across inconsistent. I list some examples:
a. The first sentences of the second results section give two distinct interpretations for the small effect of hLAMP1 expression on LASVpp entry in A549 vs. DF-1 cells. In the former case it is interpreted as indication of near optimal fusion, and in DF-1 cells as an indication of limited access of LASVpp to endosomal compartments. Can the efficiency of fusion for internalized particles be measured in the two contexts? Or alternatively, could the efficiency of internalization in the two cell lines be compared (one supplement shows this for DF-1 cells, but how about in A549?). Furthermore, it was not clear what was meant by the limited access until much later in the manuscript where it was explained that it refers to the efficiency of endocytosis. An alternative would be to move any speculation to the Discussion section.

Response: We concur with the reviewer’s comment. Accordingly, we have minimized the amount of discussion within the Results section of our revised paper. What we meant by limited access was not internalization that is rather inefficient (new Fig. S8), but rather virus trafficking to the hLAMP1-enriched endosomes. Since this model poses great challenges for its experimental testing, we removed the speculation regarding the limited access of LASVpp to ectopically expressed hLAMP1. For this reason, we did not pursue additional measurements of LASVpp uptake by A549 cells proposed by the reviewer.

b. At the end of the second Results section the authors speculate on the reasons for the discordance between fold-enhancement of forced fusion vs. infection in hLAMP1 expressing A549 cells might be due to the less efficient infection following fusion at the plasma membrane. Why would the same effect not be observed for DF-1 cells?

Response: Multiple cell type-dependent factors can contribute to the post-fusion steps of HIV-1 infection. We therefore speculate that HV-1 infection following fusion at the surface of DF-1 is more favored than in A549 cells.

2) Most fusion and infection figures report only fold-changes. To better evaluate the data, it would be important to also include absolute values perhaps as a Supplement.

Response: We appreciate this suggestion and included no-normalized data in Fig. S2 (related to Fig. 2) and Fig. S4C and D.

3) From the presented data I am not convinced of the advantage of DF-1 cells over A549 cells for the detailed kinetic dissections of hLAMP1 effects in live-cell tracking experiments.
a. The very similar relative effect of hLAMP1 expression on endosomal fusion by LASVpp in A549 and DF-1 cells is surprising even in the context of the speculated lower internalization efficiency. Even if a much smaller fraction of total particles are internalized into DF-1 vs. A549 endosomes, going from no hLAMP1 to about endogenous levels of hLAMP1 would be expected to have a much larger fold change than in the case of going from having hLAMP1 to having more of it. The relative change in the measured function should not be dependent on the efficiency of internalization (which does not change with hLAMP1 expression).

Response: We concur with the reviewer. As stated above, we removed the speculation regarding the internalization efficacy.

b. Since there are no marked differences in the effects of hLAMP1 (over)expression in the two cell lines (and given the comment under ‘a’ above), I see no advantage of choosing DF-1 cells over the more native target. It would be important to corroborate the detailed mechanistic interpretations for the role of hLAMP1 in pore expansion in A549 cells. This would be helpful both in endosomes (same magnitude of effects in DF-1 and A549) and on the plasma membrane (since endogenous LAMP1 would not be present there just the same).

Response: That’s a good point. In response to this critique, we added a new Figure S7 presenting single virus tracking results in A549 cells. Consistent with our previous published findings, LASVpp underwent type II fusion in these cells. Importantly, the same three pore dilation phenotypes – quick dilation, delayed dilation and no mCherry release – were observed in A549 cells, and the relative fractions of these 3 phenotypes were comparable to those in DF-1 cells (Figs. 4 and 5). Thus, no obvious differences in fusion pore dynamics were detected in the two cell lines, validating the use of DF-1 cells for single particle fusion experiments. Most importantly, the primary reason for focusing on DF-1 cells is that these cells, but not A549 cells, tolerated a prolonged (1 hour) exposure to pH 5.0 which is required to initiate and follow forced fusion with the plasma membrane. We apologize for neglecting to mention this in the original submission.

4) Instant velocity plots are shown in various figures but not mentioned or described in the text.

Response: Apologies. We have now included a reference to velocity plots in the text (top of page 16).

**Minor issues**:

1) On page 11, second paragraph, it should be clearly stated that the single LASVpp fusion being imaged is endosomal fusion at that stage.

Response: Addressed. Thank you.

2) Page 12, line 14, it is not clear which possibility is argued against.

Response: We agree and have edited the text to clarify this point.

3) Page 14, line 13, it is commented that Fig. 6 shows efficient single LASpp fusion. It is not clear how a couple of particles report on efficiency.

Response: We agree and have modified the sentence accordingly: “Low pH promoted efficient single LASVpp fusion with control and hLAMP1 expressing cells (exemplified in Fig. 6B, C).”

4) Please make clear in the text when soluble LAMP1 was added to virus particles relative to the attachment step of viruses to cells.

Response: sLAMP1 was added to particles pre-bound to cells. This is now stated in the text.

5) Figure 2A legend, should explain that LASVpp are expressing luciferase and when luciferase signal was measured (perhaps move the intro sentences in part D to part A).

Response: Pertinent information added.

6) Fig. S6 – why does LAMP1 have ladder-like appearance?

Response: We do not have a good explanation for this, except that there are multiple differentially glycosylated bands. We have made several sLAMP1 preparations, and all behaved the same. The fact that this heterogenous mix of hLAMP1 specifically and potently accelerated LASVpp fusion suggests that our hLAMP1 preparations are functionally active.

7) In the discussion section, perhaps add more discussion of the endosomal factors that might be redundant to LAMP1.

Response: Following the reviewer suggestion, we have added a new reference for the alternative endosomal factor that may play role in arenavirus fusion.
